# Case of Chorea Sancti Viti Cured by Cuprum Ammoniacum

**Published:** 1786

**Authors:** Robert Willan

**Affiliations:** Member of the Royal College of Physicians, and Physician to the Finsbury and Public Dispensaries in London


XIII. *Case of Chorea Sancti Viti cured by Cuprum Ammoniacum. By* Robert Willan, *M. D. Member of the Royal College of Physicians, and Physician to the Finsbury and Public Dispensaries in London.*

R. WENN, of St. John's Square, at the age of three years, had a violent fever, attended with great pain in his head and stomach, and tense painful swellings of his feet and ancles, which continued upwards of a month. About two years after, he had another attack of a similar fever, when the swellings and pain remained upon him for six weeks, leaving him at length in a very languid and debilitated state. The chorea sancti viti almost immediately succeeded this disorder, affecting his hands first, and soon after his feet, in such a manner, that he was unable to stand or assist himself in any respect.

The cold bath and various tonic remedies were directed for him by a judicious physician, but afforded no relief. He afterwards tried sea bathing with somewhat better effect: it restored the use of his lower extremities in a short time, and strengthened his hands considerably within six months.

In

In this ſtate he remained for about two years, went to ſchool, and was able, at length, to write with tolerable ſteadineſs.

About the beginning of November, 1785, the chorea ſancti viti came on ſuddenly, without any aſſignable cauſe, and to a greater degree than at firſt.

I viſited him November 25th, and found the muſcles of his face, as well as thoſe of the upper and lower extremities, generally affected with irregular convulſions. His legs were obliged to be tied together very cloſely, and his arms pinioned to his ſide, otherwiſe the motion fatigued him exceedingly. He took a laxative draught on this day, and then began with the following pills *:

℞. Cupri ammoniacal. gr. j.

Theriac. andromach. q. ſ.

Fiat pilula hor. 11[a] mat[a]. iterum veſperi ſumenda.

On the 27th the doſe of the cuprum amm. was increaſed to gr. ij. and did not diſagree with his ſtomach.—The day following he began to take gr. iij. of the cuprum amm. in two ſmall

* He had been put upon a courſe of bark and chalybeates, and continued it a week or ten days without effect.

pills, pills, and, before night, could walk about the room, though not with ſteadineſs; nor were the motions of his hands yet abated. The medicine, in the above doſe, always occaſioned a little ſickneſs; it was therefore not increaſed farther.

Nov. 29, he recovered the full uſe of his legs, and his hands were more ſteady.

Dec. 1, his hands were ſo much better, that he could write eaſily. He uſed the medicine about a week longer, and then went into the country perfectly recovered.

